# Editorial: The impact of intelligent technologies on the future of work: challenges and opportunities for individuals, organizations, and society

**DOI:** 10.3389/fsoc.2026.1877636

**Published:** 2026-06-11

**Authors:** Atif Sarwar, Lucill Curtis, Andrea Oltramari, Muhammad Siddique

**Affiliations:** 1 University of Liverpool Management School, University of Liverpool, Liverpool, United Kingdom; 2 Norwich Business School, University of East Anglia, Norwich, United Kingdom; 3 School of Business, Federal University of Rio Grande do Sul, Porto Alegre, Brazil; 4 Liverpool Hope Business School, Liverpool Hope University, Liverpool, United Kingdom

**Keywords:** artificial intelligence, future of work (FoW), GenAI (GenAI), intelligent technologies, professional boundaries, professional work

Whilst digitalization has been progressively transforming the infrastructure of work, the current wave of intelligent technologies, including AI, ML, and GenAI, are qualitatively different from previous emphasizes. Earlier digital technologies were primarily rule following, executing the instructions given by human experts reliably. This made their outputs deterministic and traceable to human decisions. Intelligent technologies have broken that pattern by inferring rules from large volumes of data, making it difficult for any individual to reconstruct them (Chalmers et al., 2026; Murray et al., 2021; Raisch and Krakowski, 2021). This has resulted in organizations increasingly structuring around technologies, whose outputs they cannot fully explain or predict (Faulconbridge et al., 2025; Glaser et al., 2024).

These technologies are reshaping decision-making, professional work, and organizing across various industries (Chalmers et al., 2026; Spring et al., 2022). For decades, professions were insulated from this change due to their protected boundaries (Faulconbridge et al., 2024) and professional identities (Scarbrough et al., 2025), combined with a consistent gap between technology's promise and organizational readiness. This has resulted in increasing tensions between the speed of technology's evolution and the reliability of its outputs, and between promised algorithmic efficiency and the contextual judgment fundamental to meaningful work (Lebovitz et al., 2021; Pakarinen and Huising, 2025).

Further research into the impact of intelligent technologies on work and workers is required because “intelligent technologies” are not a single thing. Predictive, generative, agentic, and embodied systems operate through different logics and produce distinct organizational outcomes (Jarrahi and Glaser, 2025). The studies treating these varying technologies as singular in their impact and underlying principles, often termed

as “algorithmic management,” run the risk of misrepresenting potential threats and benefits of these technologies (Kellogg et al., 2020). Different technologies elicit specific forms of adaptation, resistance, and contestation among professional workers who use them in their everyday work (Glaser et al., 2021; Pachidi et al., 2021).

These human responses are important and need studying as professionals using these technologies are not passive recipients of algorithmic change; they contest, resist, and often work around the structures placed by intelligent technologies, defending their professional autonomy and developing approaches to calibrate AI output, against their own judgment (Sarwar and Faulconbridge, 2026; Lebovitz et al., 2022). Furthermore, these micro-level dynamics cannot be divorced from macro-level institutional forces that shape how these intelligent technologies are aligned with the values of those deploying and using these systems (Glaser et al., 2025; Zuboff, 2019).

The papers in the Research Topic highlight this entanglement of macro and micro, and the social, the organizational, the professional and the technical. We believe this is a timely issue as the impact of intelligent technologies on work

“*increasingly challenges the adaptive capacity of existing organization and management theory to make sense of it*.” (Chalmers et al., 2026, p. 2)

Understanding the impact of intelligent technologies on the work reconfiguration is a pressing question facing organizations, workers and society (Anthony et al., 2023; Chalmers et al., 2026).

The first paper in the Research Topic (Nastasa et al.) argues that intelligent technologies are not just shaping the labor market, but also reconfiguring the environment for AI expertise itself. Authors, analyze around 800 job postings on EURAXESS platform, using text mining and Latent Dirichlet Allocation topic modeling. They illustrate an increasing demand for research and digital skills, along with communication and enterprise, representing distinct epistemic cultures. This contribution offers a distinctive insight into how the professional boundaries of AI work are actively reproduced through recruitment practices.

The second paper in the Research Topic (Wawera et al.) argues intelligent technology driven innovations does not occur in a vacuum, rather they are accompanied by the reconfiguration of work itself. They conducted a scoping review of 99 articles and present an integrated framework organizing workplace transformations, across four dimensions, i.e., flexibility, digitalisation, democratization, and agility, and further 14 subdimensions. Their framework offers a valuable and timely insight into the organizational conditions within which intelligent technologies are adopted, contested, and embedded.

The (Klowait and Erofeeva) paper, draws on Goffman's dramaturgical framework, providing a nuanced and sociologically grounded account of the impact of AI on work. They suggest the entanglement of AI and professionals is as symbolic as it is interactional, where workers continuously stage, conceal, and renegotiate genuine expertise. They argue that large language models, while quietly taking over the invisible, routinized labor, leave professionals enacting the visible, relational performances central to professional prestige. Interestingly, the paper was intentionally written with the aid of frontier AI models, thus making it a meta-reflexive performance of the very dynamic it theorizes. Therefore, the paper explains why utopian and dystopian narratives misread how AI affects professional work and how automation unfolds, demanding a more human-centered policy response.

The next paper (Sha and Chai) explores the employee reaction when AI is introduced into their work setting. Drawing on the Conservation of Resources Theory, they show how professionals, in response to AI, reshape (job crafting) their own roles and tasks. Surveying 370 employees actively using AI, this study highlights the mediating role, job insecurity plays in job crafting. They argue the mediating role of job insecurity is amplified or dampened by the employees' AI knowledge, thus suggesting that organizations should cultivate AI literacy rather than rely on stress as a motivational lever. The findings directly contribute to the discussion on ethical people management in the age of algorithmic transformation.

The final paper in the Research Topic (Luwei and Huimin) focuses on how intelligent technologies are not just reconfiguring tasks and processes but also professional careers. Adopting a mixed methods approach, this paper investigates the key drivers and barriers to career development, primarily in intelligent technologies-adjacent emerging industries. They argue that in these times of technological transformation, career motivation and social networks play a crucial role in instilling greater resilience in professionals as they face external career barriers, such as the introduction of intelligent technologies into the workplace. The findings highlight the importance of fair and inclusive labor market policies to support professionals in adapting to changing work environments.

## References

[B1] Anthony C. Bechky B. A. Fayard A.-L. (2023). “Collaborating” with AI: taking a system view to explore the future of work. Organ. Sci. 34, 1672–1694. doi: 10.1287/orsc.2022.1651

[B2] Chalmers D. Hunt R. Pachidi S. Potočnik K. Townsend D. (2026). The acceleration of artificial intelligence: Rethinking organization and work in an era of rapid technological change. J. Manag. Stud. 63, 285–314. doi: 10.1111/joms.70063

[B3] Faulconbridge J. Sarwar A. Spring M. (2025). How professionals adapt to artificial intelligence: the role of intertwined boundary work. J. Manag. Stud. 62, 1991–2024. doi: 10.1111/joms.12936

[B4] Faulconbridge J. R. Sarwar A. Spring M. (2024). Accommodating machine learning algorithms in professional service firms. Organ. Stud. 45, 1009–1037. doi: 10.1177/01708406241252930

[B5] Glaser V. L. Pollock N. D'Adderio L. (2021). The biography of an algorithm: performing algorithmic technologies in organizations. Organ. Theory 2, 1–27. doi: 10.1177/26317877211004609

[B6] Glaser V. L. Sloan J. Gehman J. (2024). Organizations as algorithms: a new metaphor for advancing management theory. J. Manag. Stud. 61, 2748–2769. doi: 10.1111/joms.13033

[B7] Glaser V. L. Moser C. D'Adderio L. Hannigan T. R. Pozner J.-E. (2025). Special issue on algorithmic organizing. Organ. Stud. https://journals.sagepub.com/page/oss/call-for-papers/algorithmic-organizing (Accessed April 18, 2026).

[B8] Jarrahi M. H. Glaser V. L. (2025). “Not all AI systems are created equal: a typology of AI systems' performance and affordance,” in Proceedings of the 58th Annual Hawaii International Conference on System Sciences, 7–10 January (Waikoloa, HI).

[B9] Kellogg K. C. Valentine M. A. Christin A. (2020). Algorithms at work: the new contested terrain of control. Acad. Manage. Ann. 14, 366–410. doi: 10.5465/annals.2018.0174

[B10] Lebovitz S. Levina N. Lifshitz-Assaf H. (2021). Is AI ground truth really “true”? The dangers of training and evaluating AI tools based on experts' know-what. MIS Q. 45, 1501–1525. doi: 10.25300/MISQ/2021/16564

[B11] Lebovitz S. Lifshitz-Assaf H. Levina N. (2022). To engage or not to engage with AI for critical judgments: how professionals deal with opacity when using AI for medical diagnosis. Organ. Sci. 33, 126–148. doi: 10.1287/orsc.2021.1549

[B12] Murray A. Rhymer J. Sirmon D. G. (2021). Humans and technology: forms of conjoined agency in organizational routines. Acad. Manage. Rev. 46, 552–571. doi: 10.5465/amr.2019.0186

[B13] Pachidi S. Berends H. Faraj S. Huysman M. (2021). Make way for the algorithms: symbolic actions and change in a regime of knowing. Organ. Sci. 32, 18–41. doi: 10.1287/orsc.2020.1377

[B14] Pakarinen P. Huising R. (2025). Relational expertise: what machines can't know. J. Manag. Stud. 62, 2053–2082. doi: 10.1111/joms.12915

[B15] Raisch S. Krakowski S. (2021). Artificial intelligence and management: the automation–augmentation paradox. Acad. Manage. Rev. 46, 192–210. doi: 10.5465/amr.2018.0072

[B16] Sarwar A. Faulconbridge J. (2026). “AI-professional hybrid in professional service firms: fear, hope and the rise of new professional roles,” in Hybrid Working and AI: The Impact on Organisational Development, eds. J. Randall, and B. Bernard (New York, NY: Routledge), 89–104. doi: 10.4324/9781003647843-8

[B17] Scarbrough H. Chen Y. Patriotta G. (2025). The AI of the beholder: intra-professional sensemaking of an epistemic technology. J. Manag. Stud. 62, 1885–1913. doi: 10.1111/joms.13065

[B18] Spring M. Faulconbridge J. Sarwar A. (2022). How information technology automates and augments processes: insights from artificial-intelligence-based systems in professional service operations. J. Oper. Manag. 68, 592–618. doi: 10.1002/joom.1215

[B19] Zuboff S. (2019). The Age of Surveillance Capitalism: The Fight for a Human Future at the New Frontier of Power. New York, NY: PublicAffairs.

